# Standardized clinical examination of maxillofacial injury: fracture diagnostic accuracy across experience levels

**DOI:** 10.1007/s00068-025-02927-2

**Published:** 2025-07-07

**Authors:** Pieter Date van der Zaag, Romke Rozema, Inge H.F. Reininga, Baucke van Minnen, REDUCTION study group members

**Affiliations:** 1 Department of Oral and Maxillofacial Surgery, University Medical Center Groningen, University of Groningen, Groningen, The Netherlands; 2 Department of Surgery, University Medical Center Groningen, University of Groningen, Groningen, The Netherlands; 3 Department of Trauma Surgery, University Medical Center Groningen, University of Groningen, Groningen, The Netherlands; 4 Northern Netherlands Trauma Registry, Emergency Care Network Northern Netherlands (AZNN), Groningen, The Netherlands

**Keywords:** Maxillofacial injury, Physical examination, Emergency room visits, Maxillofacial injury, Midfacial injury, Mandibular injury, Clinical examination, Physician experience

## Abstract

**Purpose:**

To determine whether physicians can accurately assess maxillofacial injury patients for the presence of fractures using a standardized clinical examination protocol, and to evaluate differences in assessment accuracy across experience levels.

**Method:**

A prospective observational cohort study was conducted in four hospital emergency departments of patients with maxillofacial injuries. Physicians rated fracture probability twice on a 0–10 scale: first based on visual signs *before* the clinical examination, and again *after* the standardized clinical examination consisting of 15 midfacial or 14 mandibular parameters. The assessors were categorized as medical intern in training, junior resident, senior resident, or consultant. The patients were classified into four groups: midfacial or mandibular injury, with or without a fracture, as determined by radiographic results. The fracture probability ratings before and after the clinical examination were compared.

**Results:**

Of 556 patients, 499 (90%) had midfacial injuries and 57 (10%) mandibular injuries. For midfacial injuries, greater variability in probability ratings and more incorrect assessments were observed compared to mandibular injuries. The intra-assessor analysis of fracture probability ratings before and after the clinical examination showed significant improvement in the direction of the eventual radiological diagnosis in three of the four patient groups for junior and senior residents, whereas the consultants, whose assessment was already proficient, only improved for one group, and none for interns. The inter-assessor analysis of fracture probability ratings showed no significant differences between the assessors.

**Conclusion:**

Midfacial injuries are more difficult to assess than mandibular injuries, and junior and senior residents benefited the most from the standardized clinical examinations.

**Supplementary information:**

The online version contains supplementary material available at 10.1007/s00068-025-02927-2.

## Introduction

Maxillofacial injuries are one of the most common types of trauma presented at the emergency departments (ED) [1, 2]. Common etiologies of maxillofacial injuries include falls, violence and road traffic accidents [3, 4]. Several studies show that the number of patients presenting at ED with maxillofacial injuries is increasing among the ageing population [5, 6]. Similarly, the incidence of maxillofacial fractures in the ED is also increasing [5–7]. Maxillofacial fractures are associated with substantial morbidity, deformity, loss of function, and high treatment costs [8]. Early diagnosis or ruling out of maxillofacial fractures in patients presenting at the ED with maxillofacial injuries is therefore essential.

Currently, the assessment of all trauma patients follows the standardized Advanced Trauma Life Support (ATLS) method, which includes ‘primary assessment’, ‘secondary assessment’ and ‘tertiary assessment’. Primary assessment focuses only on immediately life-threatening cases requiring immediate treatment [9]. Secondary assessment involves a full clinical examination, including maxillofacial injuries [10]. However, currently, no standardized clinical assessment protocol exists in the ED for maxillofacial injuries. ED physicians assess the patient, perform a risk stratification, and then consult the appropriate specialist. This presents challenges when specific maxillofacial expertise is required, as correct interpretation of clinical findings may be difficult [11]. Therefore, various decision aids for maxillofacial injuries have been proposed to support fracture assessment [12–17]. However, they either lack external validation, are limited to specific fracture types, or combine the midfacial and mandibular regions without distinction [18–20]. Furthermore, the ED is staffed by a variety of physicians with different levels of experience and expertise varying from intern in training, junior resident, senior resident to consultant [21]. Consequently the accuracy and effectiveness of injury assessment may be influenced by the physicians’ experience [22]. Studies show that less experienced physicians tend to order imaging more frequently and are associated with higher ED mortality rates [22–24]. However, research on the assessment of maxillofacial injuries by physicians with different levels of experience is lacking. Therefore, the aim of this study was twofold: (1) To determine whether physicians can accurately assess maxillofacial injury patients for fractures using a standardized clinical examination protocol; and (2) to determine whether there is a difference in the assessment of these injuries between physicians with different levels of experience. Our REDUCTION study group was established to investigate whether specific clinical parameters could be used to assess fractures in relation to maxillofacial injuries. The long-term goal of the REDUCTION study group is to provide emergency physicians with a structured tool for the clinical examination of maxillofacial injuries in the ED.

## Materials and methods

### Study design

The REDUCTION trial is a prospective cohort study [25, 26]. The trial was conducted in four hospitals in the north of the Netherlands between May 2018 and October 2019. The Institutional Review Board of the University Medical Center Groningen (Groningen, the Netherlands) confirmed that the Medical Research Involving Human Subjects Act did not apply (METc code 2017/249), and local feasibility was approved by all the hospitals.

### Patient population

All patients aged 18 years and older, who presented at the ED with either a midfacial or mandibular injury, were included. Maxillofacial injury was defined as injury to the midface or mandible, including injuries ranging from soft tissue abrasions to pan-facial fractures.

Patients without diagnostic imaging, with both midfacial and mandibular injuries, with a prior diagnosis of a maxillofacial fracture before the standardized clinical examination, and those for whom the assessor’s profession was not recorded, were excluded.

### Data collection

Each patient received a standardized clinical examination at the ED, conducted by emergency physicians, surgeons, or their residents or interns. Standardization was ensured through hands-on training, online instructional videos, and a bedside pocket card with the illustrated clinical parameters [25, 26]. The assessing physician completed a case report form documenting patient identifiers, the physician’s level of experience (intern in training, junior resident, senior resident, or consultant), and the registration of a midfacial and/or mandibular injury. The physician rated the probability of a fracture twice, once before and once after the standardized clinical examination, which was recorded on the assessment form. *Before* the clinical examination, a ‘first look’ probability assessment was conducted of midfacial or mandibular injuries, based solely on visual signs during the initial contact with the patient, and the primary survey, rated on a scale from 0 (low probability) to 10 (high probability), was completed. Next, a standardized clinical examination was performed, consisting of a checklist of 15 clinical parameters for midfacial injuries or 14 for mandibular injuries. *After* the examination, the physician reassessed the fracture probability, again using a 0–10 scale, incorporating the findings from the clinical examination.

The standardized clinical examination protocol for midfacial injuries consisted of assessing the presence of swelling, laceration, facial depression, peri-orbital hematoma, raccoon eyes, epistaxis, subconjunctival hemorrhage, ocular movement limitation, diplopia, infra-orbital nerve paresthesia, subjective malocclusion, objective malocclusion, mobility or avulsion of maxillary teeth, palpable bony step-off and maxillary mobility. Assessments of these clinical parameters were scored as either present or absent, with an additional option of “not testable” if a clinical parameter could not be assessed. For example, if there was severe swelling and/or hematoma, or if the patient’s level of consciousness prevented active patient instruction.

The standardized clinical examination protocol for mandibular injuries consisted of assessing the presence of swelling, extra-oral laceration, jaw movement pain, mouth opening limitation, inferior alveolar nerve paresthesia, intra-oral hematoma, intra-oral laceration, palpable bony step-off, mobility or avulsion of mandibular teeth, subjective malocclusion, objective malocclusion, angular compression pain, axial chin pressure pain, and the tongue blade bite test. Assessments of these clinical parameters were scored as either present or absent, with an additional option of “not testable” if a clinical parameter could not be assessed.

Patient characteristics such as sex, age, mechanism of trauma, and type of maxillofacial fracture, were taken from the electronic patient records.

### Outcome measures

The primary outcome was the presence or absence of a midfacial or mandibular fracture, as verified through orthopantomography (OPT), cone beam computer tomography (CBCT), or computer tomography (CT) scans. Midfacial fractures included fractures of the frontal sinus, orbital rim and walls, maxillary sinus, zygomaticomaxillary complex, naso-orbito-ethmoid (NOE) complex, nasal bone, Le Fort I, II, III complex, and maxillary dentoalveolar complex. Mandibular fractures were defined as any fracture involving the (para)symphysis, body, angle region, ramus, coronoid process, and condylar region, along with fractures of the mandibular dentoalveolar complex.

As a secondary outcome, the severity of the maxillofacial injury was assessed. The Facial Injury Severity Scale (FISS) was used for this purpose [27]. All maxillofacial injuries reported in the electronic patient record were scored using the FISS. The cumulative FISS score of all maxillofacial injuries per patient provides an indication of the overall severity of the maxillofacial injury. A FISS score of 0 indicates the absence of fractures, and higher scores indicate more severe maxillofacial injury with one or more fractures.

### Statistical analysis

Data analysis was performed using the Statistical Package for the Social Sciences (IBM Corp. Released 2024, IBM SPSS Statistics for Windows, Version 28.0). Categorical variables were reported as frequencies and percentages. Continuous variables were presented as means and standard deviation (SD) for normally distributed variables, and as medians with interquartile range (IQR) for non-normally distributed variables. Pearson’s χ2 test or Fisher’s exact test were used to test the differences in patient and injury characteristics between the mandibular and midfacial injury groups. The Mann-Whitney U test was used to test differences in the non-normally distributed data, such as age and FISS scores.

The first aim of this study was to determine whether physicians can assess maxillofacial injuries accurately for fractures by means of a standardized clinical examination. This was corroborated by imaging results which subsequently led to two midfacial and mandibular injury patient subgroups: one with and one without fractures. The total and intra-assessor (intern in training, junior resident, senior resident and consultant) differences in fracture probability ratings *before* and *after* the clinical examination were tested using the Generalized Linear Model (GLM) repeated measures analysis for normally distributed data with a large sample size. Regarding a non-normal distribution or small sample size, the Wilcoxon signed-rank test was used to assess these differences.

The second aim of this study was to determine whether there was a difference in the assessment of midfacial and mandibular injuries, both *before* and *after* a standardized clinical examination, between assessors with different levels of experience. The same four subgroups were analyzed: midfacial and mandibular injuries, with and without fractures. Inter-assessor (between the intern in training, junior resident, senior resident and consultant) differences in fracture probability ratings before and after the clinical examination were tested using ANOVA with Bonferroni correction for normally distributed data from a large sample size. A Kruskal-Wallis H test with Bonferroni correction was used to assess these differences in the non-normal distribution or small sample size cases. Noteworthy is that patients with more severe maxillofacial injuries involving multiple maxillofacial fractures are generally easier to diagnose for fractures. Moreover, potentially severe injuries are assigned more often to experienced physicians in the ED. To assess such a potential source of bias, the statistical tests were also applied to the FISS scores to determine if maxillofacial injury severity is associated with the assessors’ experience.

## Results

### Patient characteristics

A total of 1018 patients presented at the ED with maxillofacial injuries between May 2018 and October 2019. For this study, 128 patients were excluded from because no radiographic images were made of their maxillofacial injury. Moreover, 271 patients were excluded for having both midfacial and mandibular injuries, 61 patients for having a fracture diagnosed prior to the rating and, regarding two patients, the assessor’s level of experience was not recorded. As a result, 556 patients’ data were available for further analysis. The patients’ characteristics are presented in Table 1.

**Table 1:** Patient characteristics of the total study population, and of the midfacial or mandibular injury groups

	Total	Midfacialinjury	Mandibular injury	*p*-value
Patients n (%)	556 (100)	499 (90)	57 (10)	
Female sex n (%)	283 (51)	263 (53)	20 (35)	0.01*
Age in years (median (IQR))	58 (40)	62 (35)	33 (26)	<0.001*
Trauma mechanism n (%)				
ADL at home	189 (34)	175 (35)	14 (24)	0.14
Work	14 (3)	12 (2)	2 (3)	0.64
Traffic	234 (42)	212 (42)	22 (39)	0.09
Sport	19 (3)	17 (3)	2 (3)	0.99
Violence	69 (12)	54 (11)	15 (26)	0.001*
Fall	1 (<1)	1 (<1)	0 (0)	1.00^c^
Suicide attempt	2 (< 1)	2 (2)	0 (0)	1.00^c^
Otherwise	21 (4)	19 (4)	2 (3)	0.88
Not verifiable	7 (1)	7 (1)	0 (0)	0.36
Fracture present n (%)				
Yes	219 (39)	187 (37)	32 (56)	0.006*
No	337 (61)	312 (63)	25 (44)	
FISS median (IQR)^a^	0 (1)	0 (1)	1 (3)	<0.001*
FISS median (IQR)^b^	1 (1)	1 (0)	3 (1)	<0.001*
Profession of the assessor n (%)				
Intern in training	16 (3)	15 (3)	1 (2)	0.62^c^
Junior resident	340 (61)	309 (62)	31 (54)	<0.001*
Senior resident	117 (21)	100 (20)	17 (30)	<0.001*
Consultant	83 (15)	75 (15)	8 (14)	<0.001*

*p<0.05; ^a^ Total population; ^b^ Population with a fracture; ^c^ Fisher’s exact test performed

*IQR*; Interquartile Range, *ADL*; Activities of Daily Living, *FISS*; Facial Injury Severity Scale

Out of these 556 patients, 499 (90%) had midfacial injuries and 57 (10%) had mandibular injuries. The midfacial injury group included more women (53% vs. 35%, *p*= 0.01) and was older than the mandibular injury group (median age 62 years versus 33 years, respectively, *p* < 0.001). The mandibular injury group had a higher incidence of violence as a trauma mechanism (26% vs. 11%, *p* < 0.001), a greater presence of fractures (56% vs. 37%, *p*= 0.006), and a significantly higher FISS score (median score of 1 versus 0, respectively *p* < 0.001) than the midfacial injury group. Moreover, the mandibular fracture(s) group had a significantly higher FISS score (median score of 3 versus 1, respectively, *p* < 0.001) compared to the midfacial fracture(s) patients. 

### Maxillofacial injury severity 

The FISS scores of these patients were compared to determine if there was a difference in injury severity between patients assessed by assessors with different levels of experience. Table 2 shows the FISS scores of the patients with fractures in the midfacial (*n* = 187) and mandibular (*n* = 32) injury groups, categorized per assessor. No significant differences in injury severity classification were found between the assessors with different levels of experience with respect to both midfacial injury (*p =* 0.72) and mandibular injury (*p =* 0.78).

**Table 2:** Injury severity of patients *with* maxillofacial fractures, per assessor’s level of experience

Physician group	Patients with a midfacial fracture *n*	Median FISS (IQR)	Patients with a mandibular fracture *n*	Median FISS (IQR)
Intern in training	4	1 (5)	1	2
Junior resident	113	1 (0)	17	3 (2)
Senior resident	38	1 (0)	10	2 (2)
Consultant	32	1 (0)	4	3 (2)
*p*-value		0.72		0.78

*IQR*; Interquartile Range, *FISS*; Facial Injury Severity Scale

### Maxillofacial injury assessment

**Fig. 1:**
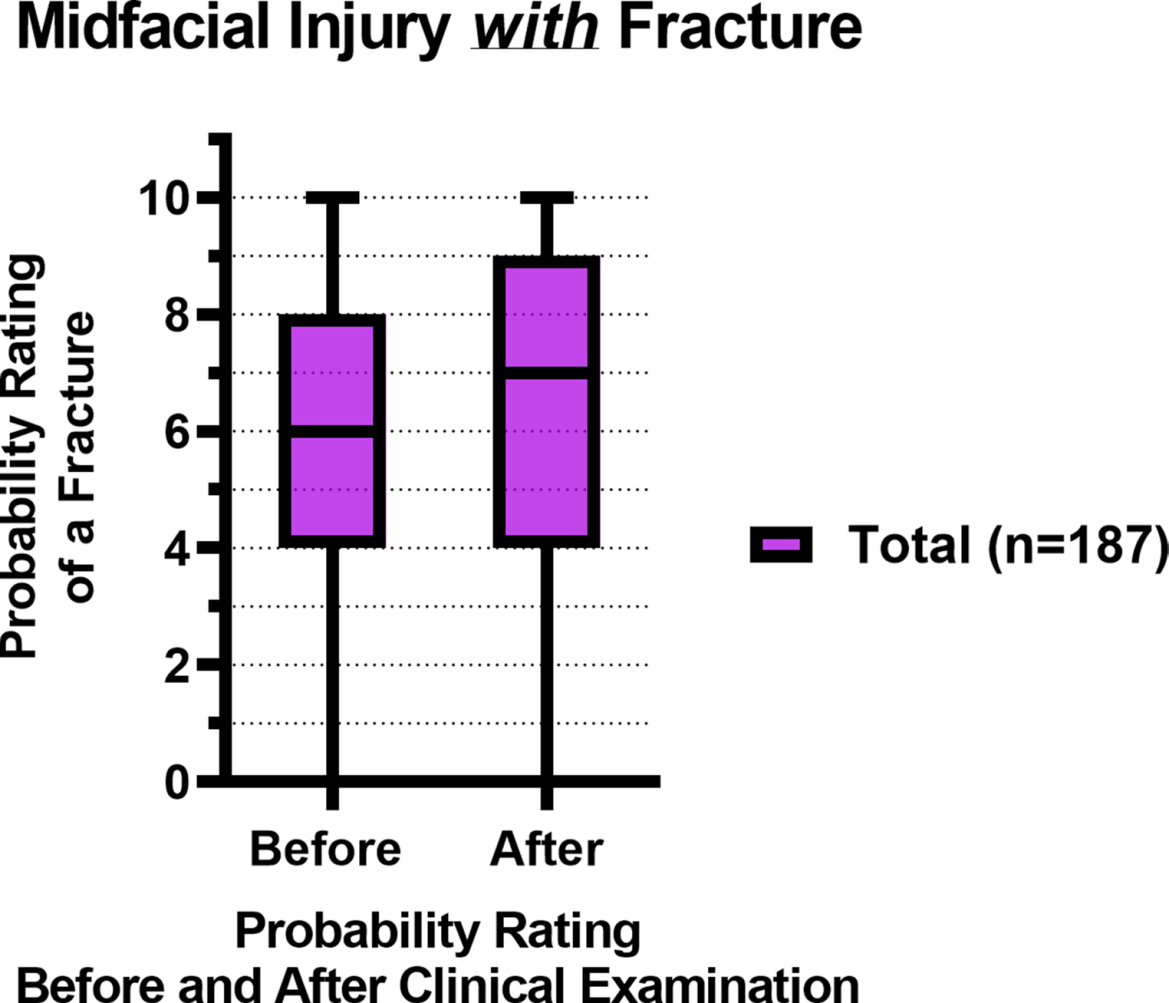
Box plots with the median and IQR of the fracture probability ratings of midfacial injury patients *with* fracture(s), before and after the clinical examination, where 0 = lowest probability of a midfacial fracture, 10 = highest probability of a midfacial fracture

Figure 1; Table 3 show the total probability rating of a midfacial fracture before and after a standardized clinical examination of patients *with* a fracture (*n* = 187). The median probability rating increased from 6 to 7 after the clinical examination (*p* < 0.001).

**Table 3:** Probability ratings of a fracture in patients with midfacial injuries

	With midfacial fracture			Without midfacial fracture		
Physician group	Median Probability Rating **Before** Clinical Examination, (IQR)	Median Probability Rating **After** Clinical Examination, (IQR)	Intra-assessor *p*-value	Median Probability Rating **Before** Clinical Examination, (IQR)	Median Probability Rating **After** Clinical Examination, (IQR)	Intra-assessor *p*-value
Total	6 (4)	7 (5)	< 0.001*	3 (3)	2 (3)	< 0.001*
Intern in Training	7 (5)	5.5 (5)	0.66	4 (6)	6 (6)	0.39
Junior Resident	6 (4)	7 (5)	0.01*	3 (4)	2 (3)	< 0.001*
Senior Resident	5.5 (4)	8 (4)	0.02*	2 (4)	2 (2)	< 0.001*
Consultant	7 (7)	7.5 (8)	0.72	3 (3)	2 (2)	< 0.001*
Inter-assessor *p*-value	0.80	0.77		0.06	0.05	

*IQR*; Inter Quartile Range, *p < 0.05

**Fig. 2:**
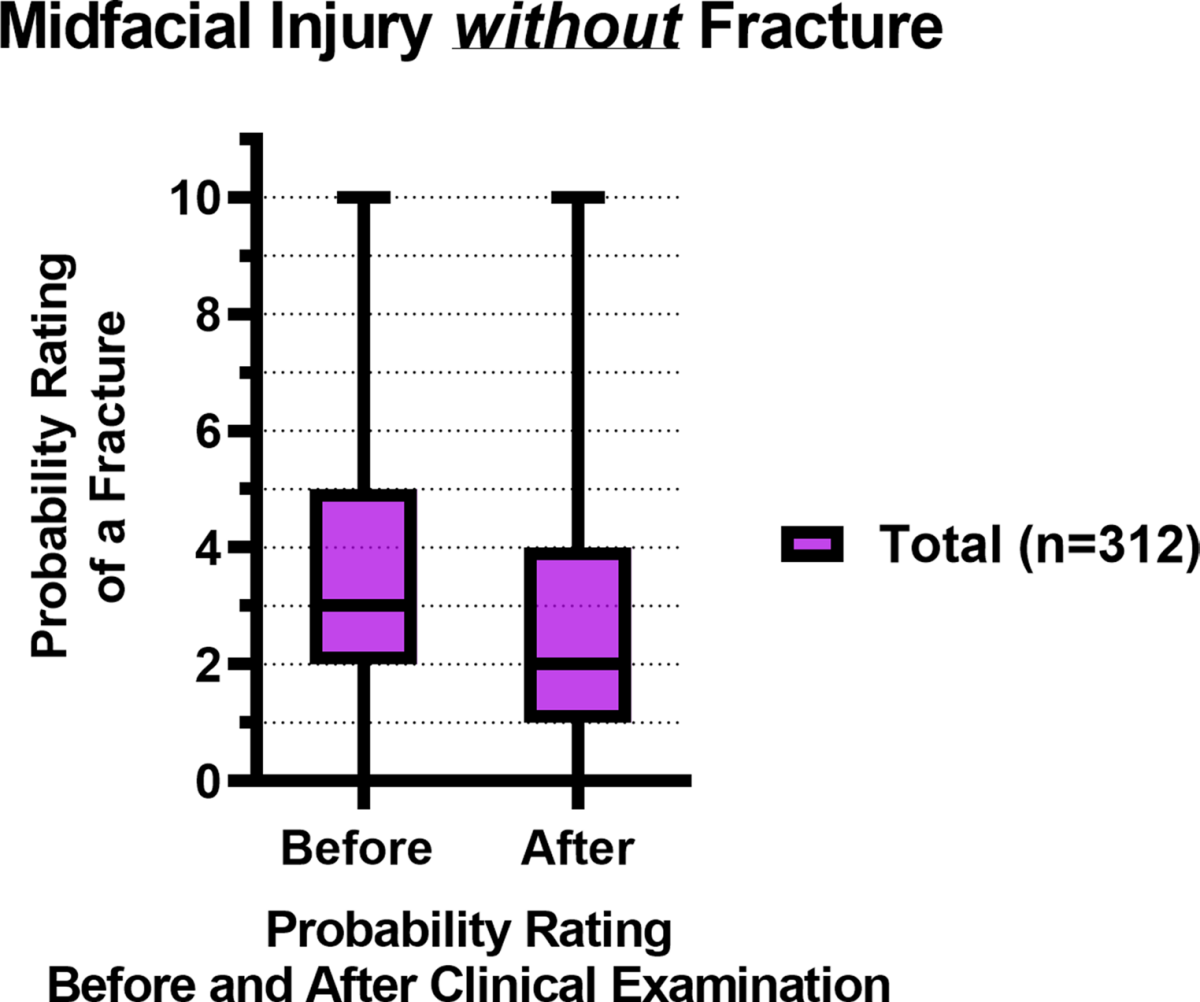
Box plots with the median and IQR of the fracture probability ratings of midfacial injury patients *without* fracture(s), before and after the clinical examination, where 0 = lowest probability of a midfacial fracture, 10 = highest probability of a midfacial fracture

Figure 2; Table 3 show the total probability ratings of a midfacial fracture before and after a standardized clinical examination of patients *without* a fracture (*n* = 312). The median probability rating decreased from 3 to 2 after the clinical examination (*p* < 0.001).

**Fig. 3:**
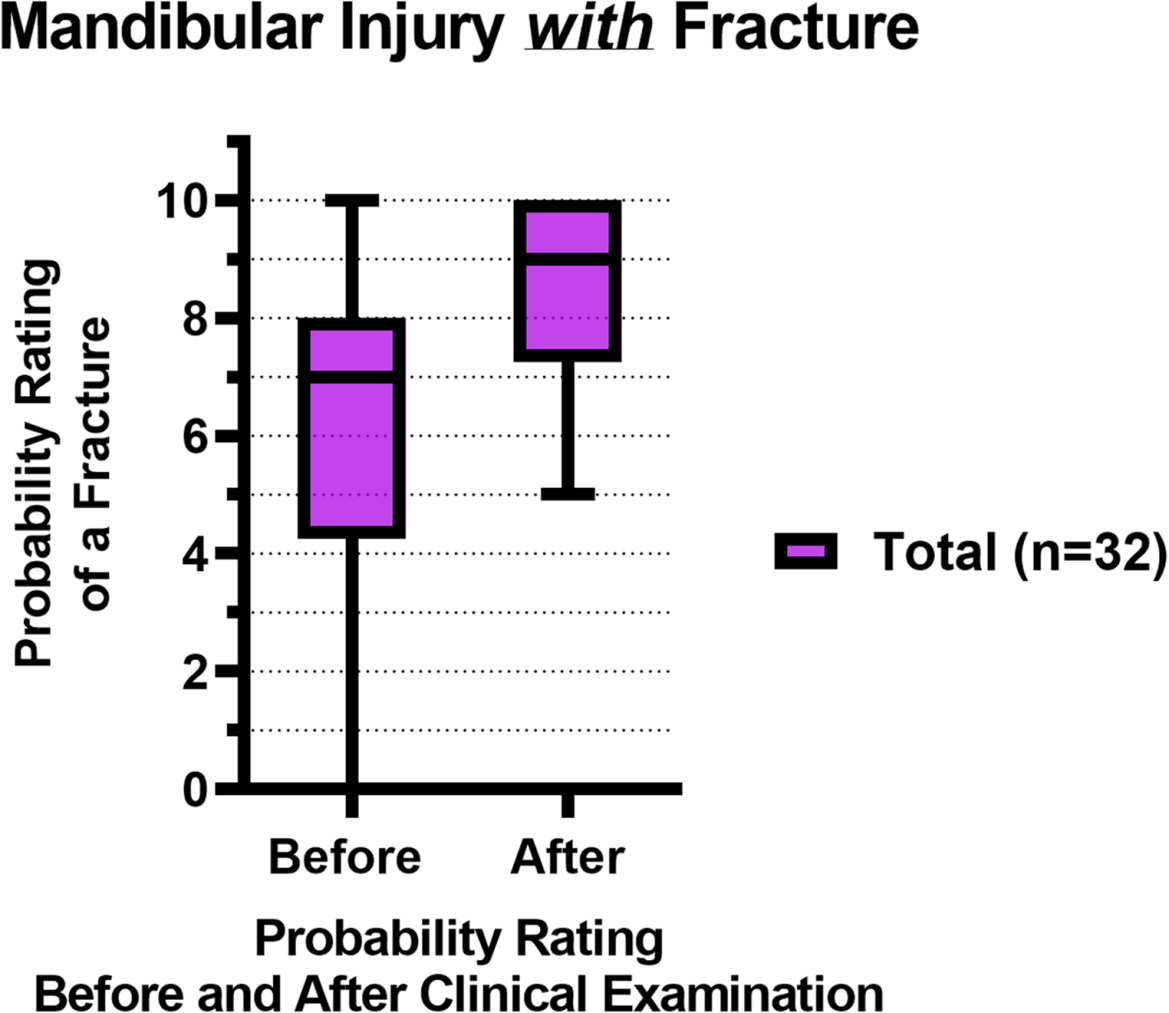
Box plots with the median and IQR of the fracture probability ratings of mandibular injury patients *with* a fracture, before and after the clinical examination, where 0 = lowest probability of a mandibular fracture, 10 = highest probability of a mandibular fracture

Figure 3; Table 4 show the total probability ratings of a mandibular fracture before and after a standardized clinical examination of patients *with* a fracture (*n* = 32). The median fracture probability rating increased from 7 to 9 after the clinical examination (*p* < 0.001).

**Table 4:** Probability ratings of a fracture in patients with mandibular injuries

	With mandibular fracture			Without mandibular fracture		
Physician group	Median Probability Rating **Before** Clinical Examination, (IQR)	Median Probability Rating **After** Clinical Examination, (IQR)	Intra-assessor *p*-value	Median Probability Rating **Before** Clinical Examination, (IQR)	Median Probability Rating **After** Clinical Examination, (IQR)	Intra-assessor *p*-value
Total	7 (4)	9 (3)	< 0.001*	2 (4)	2 (5)	0.39
Intern in Training	1	7	n/a			
Junior Resident	7 (5)	9 (3)	0.002*	2.5 (4)	2 (5)	0.84
Senior Resident	7 (3)	9.5 (2)	0.01*	2 (2)	2 (2)	0.32
Consultant	8 (6)	8.5 (4)	0.18	1.5 (2)	1 (3)	0.32
Inter-assessor *p*-value	0.32	0.69		0.51	0.40	

*IQR*; Inter Quartile Range, *p < 0.05, n/a; Not Applicable

**Fig. 4:**
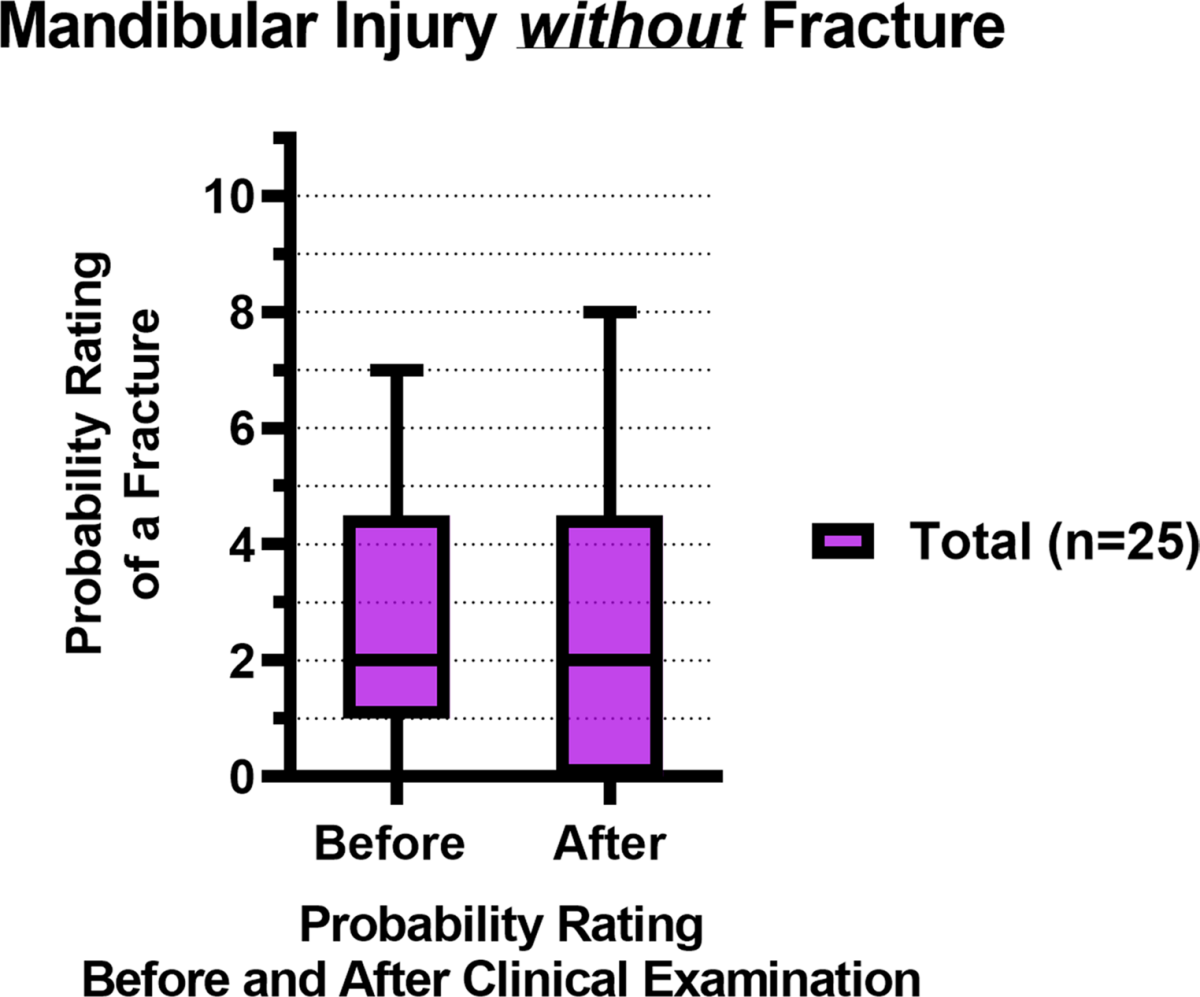
Box plots with the median and IQR of the fracture probability ratings of mandibular injury patients *without* a fracture, before and after the clinical examination, where 0 = lowest probability of a mandibular fracture, 10 = highest probability of a mandibular fracture

Figure 4; Table 4 show the total probability ratings of a mandibular fracture before and after a standardized clinical examination of patients *without* a fracture (*n* = 25). The median fracture rating remained 2 after the clinical examination (*p* = 0.39).

**Fig. 5:**
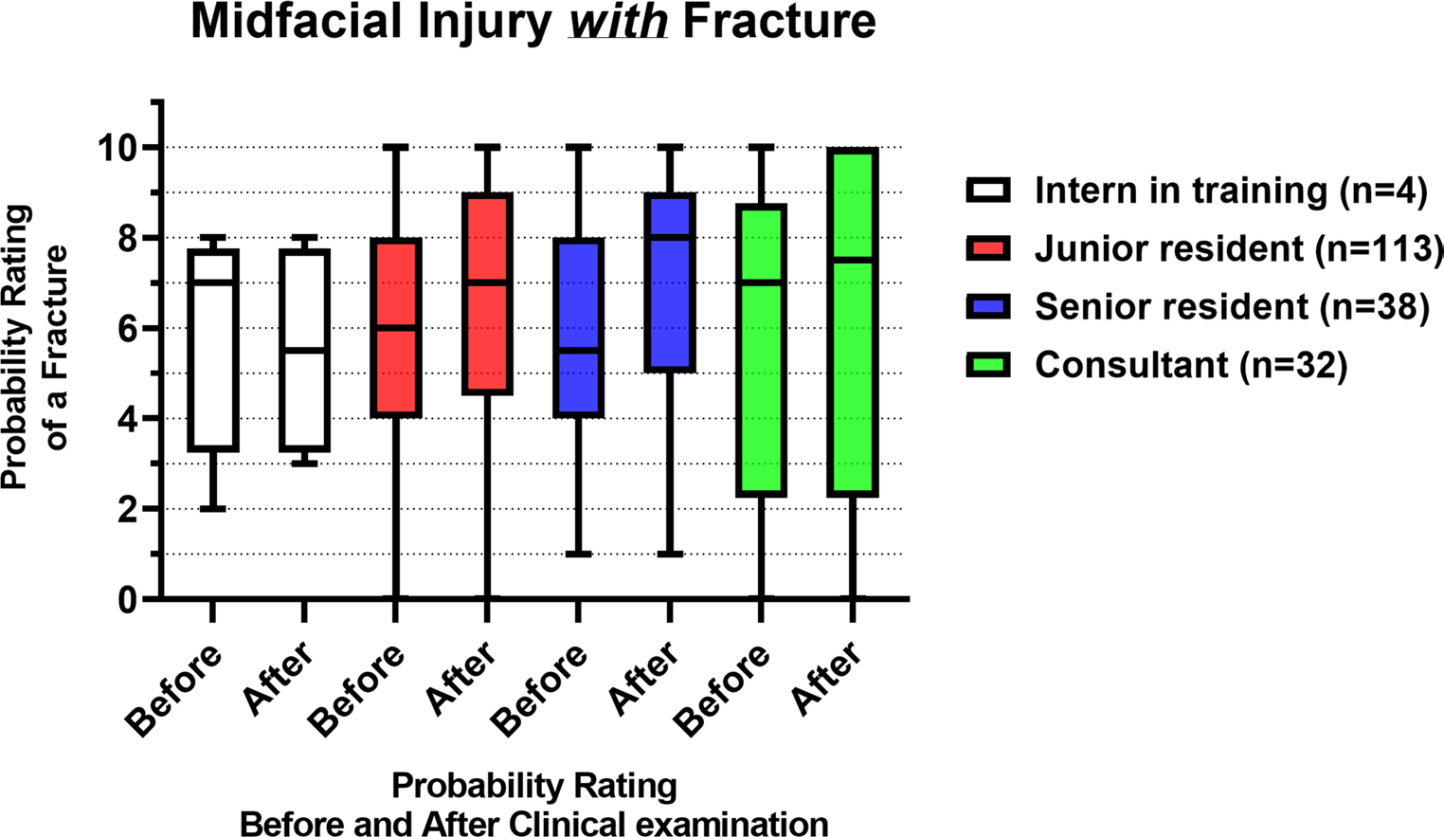
Box plots with the median and IQR of the fracture probability ratings of midfacial injury patients *with* fracture(s), before and after the clinical examination, where 0 = lowest probability of a midfacial fracture, 10 = highest probability of a midfacial fracture

Figure 5; Table 3 show the probability ratings of a midfacial fracture before and after a standardized clinical examination of patients *with* a fracture (*n* = 187). Both the junior and senior residents showed a significant difference in their probability ratings before and after the clinical examination (*p* = 0.01 and *p* = 0.002, respectively). There was no significant inter-assessor difference in probability ratings before (*p =* 0.80) and after the clinical examination (*p =* 0.77).

**Fig. 6:**
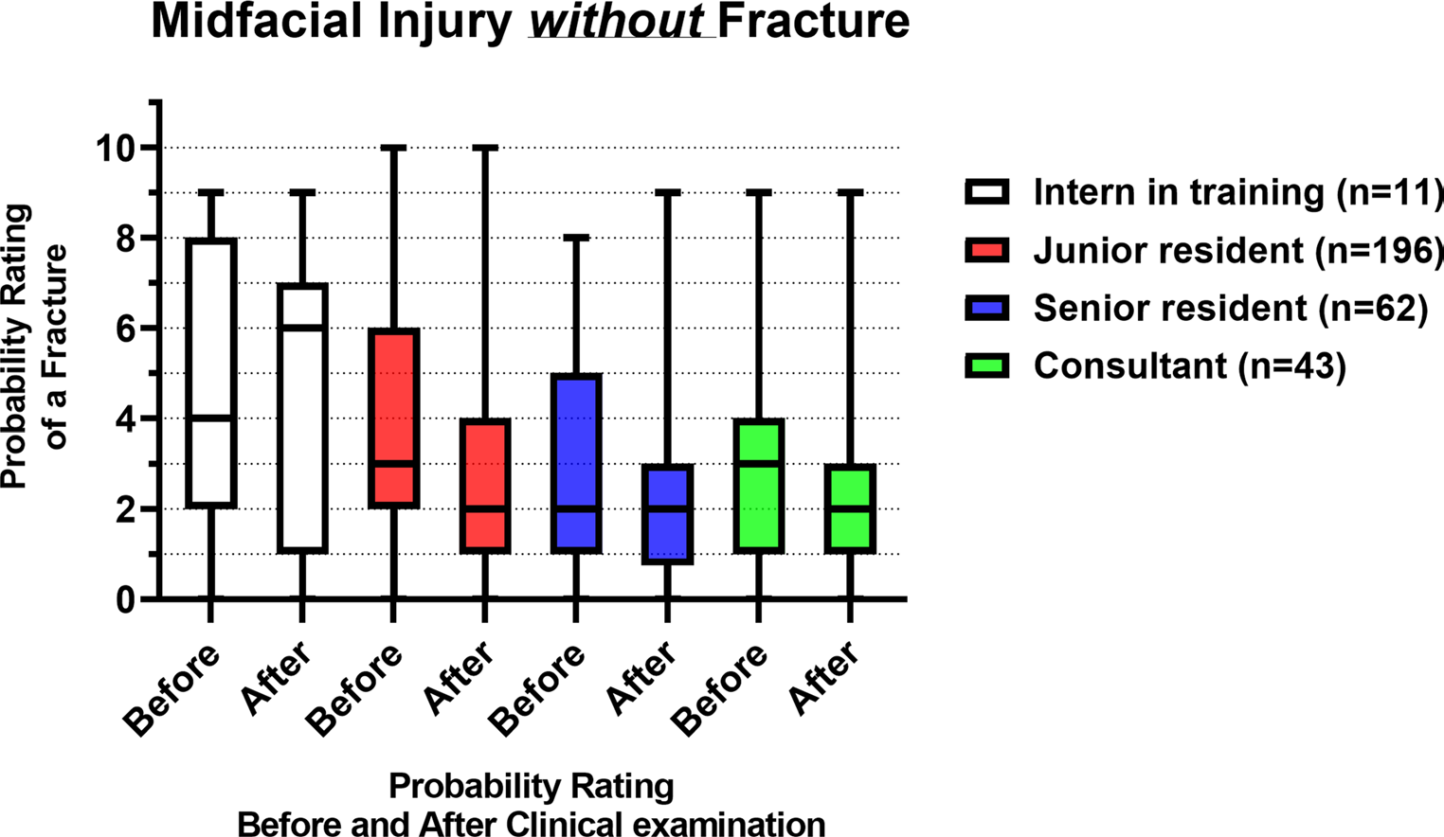
Box plots with the median and IQR of the fracture probability ratings of midfacial injury patients *without* fracture(s), before and after the clinical examination, where 0 = lowest probability of a midfacial fracture, 10 = highest probability of a midfacial fracture

Figure 6; Table 3 show the probability ratings of a midfacial fracture before and after a standardized clinical examination of the patients *without* a fracture (*n* = 312). The junior residents, senior residents and consultants showed a significant difference in their probability ratings before and after the clinical examination (*p* < 0.001, *p* < 0.001 and *p* < 0.001, respectively). There was no significant inter-assessor difference in probability ratings before (*p* = 0.06) and after the clinical examination (*p* = 0.05).

**Fig. 7:**
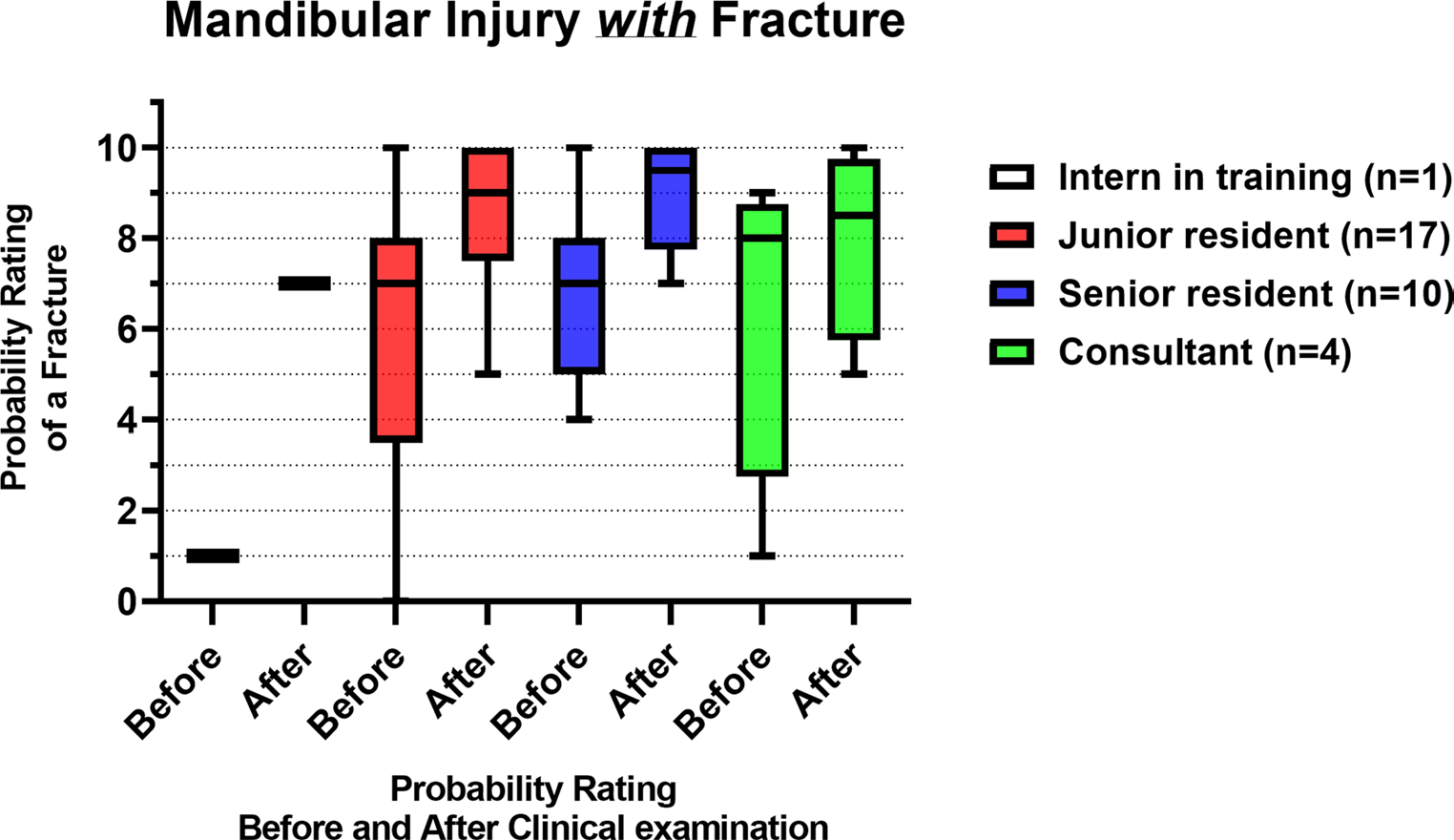
Box plots with the median and IQR of the fracture probability ratings of mandibular injury patients *with* a fracture, before and after the clinical examination, where 0 = lowest probability of a mandibular fracture, 10 = highest probability of a mandibular fracture

Figure 7; Table 4 show the probability ratings of a mandibular fracture before and after a standardized clinical examination of patients *with* a fracture (*n* = 32). Both the junior and senior residents showed a significant difference in their probability ratings before and after the clinical examination (*p*= 0.002 and *p*= 0.01, respectively). There was no significant inter-assessor difference in fracture probability ratings both before (*p*= 0.32) and after the clinical examination (*p*= 0.69).

**Fig. 8:**
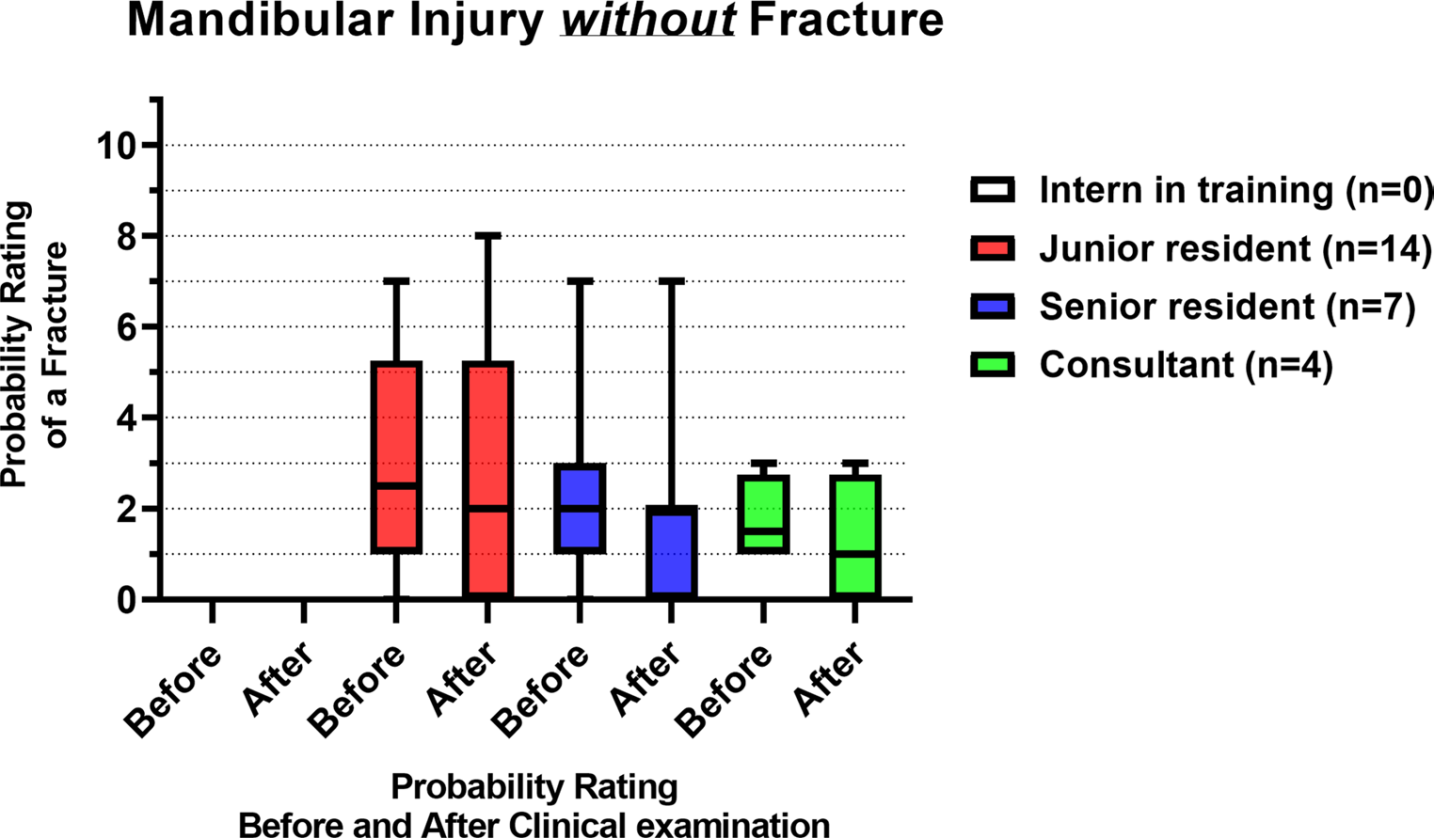
Box plots with the median and IQR of the fracture probability ratings of mandibular injury patients *without* a fracture, before and after the clinical examination, where 0 = lowest probability of a mandibular fracture, 10 = highest probability of a mandibular fracture

Figure 8; Table 4 show the probability ratings of a mandibular fracture before and after a standardized clinical examination of patients *without* a fracture (*n* = 25). There was no significant difference in probability ratings before and after the clinical examination among all the assessor groups. There was no significant inter-assessor difference in probability ratings both before (*p =* 0.51) and after the clinical examination (*p =* 0.40).

## Discussion

The first aim of this study was to determine whether physicians can accurately assess fractures in patients with maxillofacial injuries using a standardized clinical examination protocol. This study shows that a standardized clinical examination improves fracture assessment accuracy. This, in turn, can lead to more effective use of imaging, resulting in a reduction in patient radiation exposure and costs. However, a midfacial injury appears to be more challenging to assess than a mandibular injury, with a wider range of probability ratings and more incorrect assessments.

The second aim of this study was to determine whether there is a difference in the assessment of these injuries between physicians with different levels of experience. This study showed that the junior and senior residents improved the most using the standardized clinical examination protocol. Overall, however, the difference in the fracture probability ratings of midfacial and mandibular injuries was not significant between assessors with different levels of experience.

### Midfacial injury with fracture

First, it was assessed whether patients with more severe midfacial fractures were more often assigned to more experienced physicians in the ED. The median FISS score for midfacial fractures was 1 across all physician groups, indicating comparable injury severity regardless of experience level. In the population *with* midfacial fractures, the median fracture probability rating increased from 6 to 7 after the clinical examination, indicating a higher probability of a midfacial fracture. However, this increase is limited and, with a score of 7 out of 10, does not appear to be conclusive in the diagnosis of midfacial fractures. Furthermore, 0- and 1-ratings (i.e., the lowest probability of a midfacial fracture) occurred both before and after the clinical examination, indicating that completely incorrect assessments were made. These completely incorrect assessments could have been due to the overlap of midfacial anatomical structures, which is a complicating factor in the assessment of fractures in midfacial injuries. Furthemore, midfacial swelling can interfere with the assessment of clinical parameters such as facial depression, palpable bony step-off, and ocular movement limitation. Despite the limited sample size, it is noteworthy that the interns in training showed a decline in assessment accuracy with the standardized clinical examination, with the median probability rating decreasing from 7 to 5.5. This decline in accuracy may be due to interns’ limited experience in interpreting clinical findings. As a result, they may not fully appreciate the findings derived from the clinical examination. The junior and senior residents showed a significant improvement in ratings after the clinical examination, showing that the standardized clinical examination protocol improved their assessment. The consultants’ initial probability ratings were already high, possibly due to their experience, and their ratings did not really change following the clinical examination. No significant differences in fracture probability ratings were found between the assessors both before and after the clinical examination, indicating that the assessors’ ratings of midfacial fractures were similar. This suggests that physician experience does not play a critical role in the diagnosis of midfacial fractures.

### Midfacial injury without fracture

In the population *without* midfacial fractures, the median fracture probability rating decreased from 3 to 2 after the clinical examination, indicating a lower probability of a midfacial fracture. The ruling out of midfacial fractures seemed to be facilitated by the clinical examination. However, the probability ratings before the clinical examination were already quite low, i.e., accurate. Although 9- and 10-ratings (i.e., the highest probability of a fracture) were also observed both before and after the clinical examination, indicating that completely incorrect assessments occurred across all levels of experience. Similar findings were observed in patients with midfacial fractures, demonstrating that despite the use of this extensive clinical examination protocol, achieving absolute accurate clinical assessments of midfacial injuries remains difficult. The junior and senior residents, as well as consultants, showed statistically significant improvements in probability ratings for ruling out midfacial fractures. Once again, the interns in training showed a decline in assessment accuracy, with a median probability rating of 4 before, and 6 after the clinical examination. No significant differences in fracture probability ratings were observed between assessors before and after the clinical examination. However, there was a trend in more experienced assessors performing better than less experienced assessors both before and after the clinical examination. This suggests that physician experience plays a role in the ruling out of midfacial fractures.

When comparing the diagnosis and ruling out of midfacial injury fractures after the clinical examination, both the median probability rating (7 for diagnosis versus 2 for ruling out) and the interquartile range (5 versus 3, respectively) suggest that a clinical examination was more effective at ruling out midfacial fractures than at diagnosing them. This aligns with previous research, which emphasizes the higher value of absent findings in ruling out midfacial fractures, while present symptoms have limited diagnostic value for confirming them [28]. 

### Mandibular injury with fracture

First, it was assessed whether patients with more severe mandibular fractures were more frequently assigned to more experienced physicians in the ED. Interns and senior residents had a median FISS score of 2, while junior residents and consultants had a median score of 3, indicating comparable injury severity regardless of experience level. In the population *with* mandibular fractures, the median fracture probability rating increased from 7 to 9 after the clinical examination, indicating a higher probability of a mandibular fracture. Regarding the mandible, the presence of a fracture was often suspected at the first look prior to the clinical examination, and subsequently confirmed by the clinical examination. Ratings of 0- and 1 (i.e., lowest probability of a fracture) were only given before the clinical examination. No completely incorrect assessments were reported after the clinical examination, highlighting the effectiveness of an examination in diagnosing mandibular fractures. The junior and senior residents’ fracture probability ratings improved significantly after the standardized clinical examination. Statistical testing was not feasible for the interns in training due to only having a single participant, although an improvement was observed. The consultants, whose initial assessments were already high, exhibited less improvement after the clinical examination. No significant differences were found in fracture probability ratings between experience levels before and after the clinical examination, indicating that mandibular fractures were rated similarly by all the assessors. This suggests that experience does not play a role in the diagnosis of mandibular fractures.

### Mandibular injury without fracture

In the population without mandibular fracture(s), the median fracture probability rating remained 2 after the clinical examination. Regarding ruling out mandibular fractures, the absence of a fracture was apparently already evident at the first look. The standardized clinical examination did not provide any additional improvement in the fracture probability ratings. Consequently, 9- and 10-ratings (i.e., the highest probability of a fracture) were not observed, both before and after the clinical examination, indicating that completely incorrect assessments had not occurred across all physician levels. In addition, there was no significant improvement in fracture probability ratings after the examination, regardless of the physician’s level of experience. No significant differences in fracture probability ratings were found between different assessor experience levels, before and after the clinical examination, indicating that mandibular injuries were rated similarly by all the assessors. This suggests that experience did not play a critical role in the ruling out of mandibular fractures.

When comparing the diagnosis and ruling out of fractures in injured mandibles after the clinical examination, both the median probability rating (9 for diagnosis versus 2 for ruling out) and the interquartile range (3 versus 5, respectively) suggest that a clinical examination was more effective at diagnosing mandibular fractures than ruling them out. Overall, mandibular fractures seem to be easier to diagnose and exclude than midfacial fractures. This aligns with existing literature, as the symptoms of mandibular fractures are often more apparent [29]. In contrast, midfacial fractures are typically less obvious, making them more difficult to identify or exclude through a clinical examination [30]. Given these challenges, three-dimensional radiographic imaging remains necessary for assessing midfacial injuries. To optimize clinical decision-making, a clinical decision aid focusing on reliably ruling out maxillofacial fractures—particularly midfacial fractures—could help to reduce unnecessary computed tomography scans following maxillofacial trauma.

### Clinical decision aids for maxillofacial injury

The Wisconsin criteria have been proposed for ruling out facial fractures, but external validation was not achieved in other studies [12, 13, 18–20]. Several decision aids have been developed for orbital fractures [14–16]. However, their use is limited as they apply only to a specific fracture type and not to the broader population of patients with maxillofacial injuries at the ED. One study reported that a combination of malocclusion, palpable tenderness, swelling, and a positive tongue-blade bite test had 100% specificity for detecting fractures of the maxilla and mandible [17]. However, this decision aid has yet to be externally validated. Four other decision aids have been proposed: two for midfacial injury and two for mandibular injury [25, 26]. The decision aid for ruling out midfacial fractures consists of peri-orbital hematoma, epistaxis, ocular movement limitation, infra-orbital paraesthesia, palpable bony step-off, and tooth mobility or avulsion, with a sensitivity of 89.7 (86.0–92.5) and a Negative Predictive Value (NPV) of 83.9% (78.4–88.2).^25^ The decision aid for ruling out midfacial fractures requiring active treatment consists of facial depression, epistaxis, ocular movement limitation, palpable bony step-off, objective malocclusion, and tooth mobility or avulsion, with a sensitivity of 97.3 (90.7–99.3) and a NPV of 99.3% (97.3–99.8).^26^ The decision aid for ruling out mandibular fractures consists of the angular compression test, axial chin pressure, objective malocclusion, tooth mobility or avulsion, and the tongue blade bite test, with a sensitivity of 98.5 (91.9–99.7) and a NPV of 98.7% (92.8–99.8).^25^ The decision aid for ruling out mandibular fractures requiring active treatment consists of mouth opening limitation, jaw movement pain, objective malocclusion, and tooth mobility or avulsion, with a sensitivity of 100.0 (90.6–100.0) and a NPV of 100.0% (96.1–100.0).^26^ These decision aids are currently undergoing external validation [31]. 

### Limitations

This study has limitations. First, the case report form included a single fracture probability rating (0–10) rather than separate assessments per region, making it unfeasible to include patients with both midfacial and mandibular injuries. A fracture in one region but not the other could lead to conflicting scores, preventing accurate evaluations. Consequently, 271 patients were excluded. Second, the physicians’ experience levels were categorized by role — intern, junior resident, senior resident, and consultant — rather than years of experience. While experience may vary within roles, this approach does provide a structured evaluation of clinical assessment per assessor, and aligns with what occurs in clinical practice. Similarly, the small number of interns in this study likely reflects clinical practice, where interns rarely assess maxillofacial injuries during their ED rotations.

In conclusion, a standardized clinical examination improves the accuracy of fracture probability assessments in maxillofacial injury patients. Midfacial injuries are more difficult to assess than mandibular injuries, with a wider range of fracture probability ratings and more incorrect assessments. A clinical examination was more useful in ruling out midfacial fractures than in diagnosing them. In contrast, clinical examinations contributed more to the diagnosis of mandibular fractures than to ruling them out. The junior and senior residents’ assessments improved the most after the standardized clinical examination. Overall, however, there were no significant differences in fracture probability ratings between physicians with different levels of experience.

## Supplementary information

Below is the link to the electronic supplementary material.

ESM 1 (DOCX 23.1 KB)

## Data Availability

No datasets were generated or analysed during the current study.
